# Items

**Published:** 1898-07

**Authors:** 


### ITEMS.

WYATT WINGRAVE (London *Lancet*, May 7, 1898,) mentions a personal necessity that arose in the writer's experience for a reliable starch digestant. A crucial comparative examination was therefore made of many malt extracts and of taka-diastase, the tests being conducted both chemically and clinically. He summarises briefly: (1) That taka-diastase is the most powerful of the starch or diastatic ferments and the most reliable since it is more rapid in its action—viz., " it will convert a larger amount (of starch) in a given time than will any other amylolytic ferment." (2) That taka-diastase seems to be less retarded in its digestive action by the presence of the organic acids butyric, lactic, acetic and also by tea, coffee and alcohol, than are saliva and the malt extracts. This is an important point in pyrosis. (3) That all mineral acids, hydrochloric, etc., quickly stop and permanently destroy all diastatic action if allowed sufficient time and if present in sufficient quantities. (4) That taka-diastase and malt diastase have, like ptyalin, no action upon (cellulose) uncooked starch. All starch food should therefore be cooked to permit of the starch ferment assisting nature in this function.

---

WAMPOLE'S war atlas with marginal index is one of the most useful articles ever sent out by a manufacturing pharmacist to the profession. It contains the flags of the nations, in minature, a map of the world, of North America, of Europe, of Spain and Portugal, of the West Indies, of Cuba, of China and the Phillipines and of the United States. Nothing could be moie helpful to every physician at the present time, and our appreciative thanks is hereby tendered for the one sent us. The maps are made by the well-known house of Rand, McNally & Co., Chicago. The atlas can be obtained by addressing Messrs. H. K. Wampole & Co., manufacturing pharmacists, 441 Green street, Philadelphia, Pa.

---

THE Doliber-Goodale Company, of Boston, announces that it has changed its name to Mellin's Food Company of North America. There has been no change in the organisation of the company nor in its management; the change is a change of name only.

---

MESSRS. G. W. Flavell & Brother, of Philadelphia, announce that it is their desire to extend their appreciation to the medical profession of the continued favor manifested toward their line of goods, by stating that there will be no advance in prices for goods on account of any stamp war tax.

---

JOHN WYETH & BROTHER, of Philadelphia, merit full confidence as to any statement made concerning their products. We take pleasure in calling the attention of our readers to the advertisement of their solution iron and manganese peptonate. This combination ought to be a valuable agent in building up debilitated anemic conditions. As a blood-maker, tonic and general roborant, it claims the attention of the profession whenever such reconstructives are indicated.

